# The nuclear export protein exportin‐1 in solid malignant tumours: From biology to clinical trials

**DOI:** 10.1002/ctm2.1684

**Published:** 2024-05-23

**Authors:** Chuanxi Lai, Lingna Xu, Sheng Dai

**Affiliations:** ^1^ Department of Colorectal Surgery Sir Run Run Shaw Hospital School of Medicine Zhejiang University Hangzhou China; ^2^ Key Laboratory of Biotherapy of Zhejiang Province Hangzhou China

**Keywords:** cancer, drug resistance, XPO1, XPO1 inhibitor

## Abstract

**Background:**

Exportin‐1 (XPO1), a crucial protein regulating nuclear‐cytoplasmic transport, is frequently overexpressed in various cancers, driving tumor progression and drug resistance. This makes XPO1 an attractive therapeutic target. Over the past few decades, the number of available nuclear export‐selective inhibitors has been increasing. Only KPT‐330 (selinexor) has been successfully used for treating haematological malignancies, and KPT‐8602 (eltanexor) has been used for treating haematologic tumours in clinical trials. However, the use of nuclear export‐selective inhibitors for the inhibition of XPO1 expression has yet to be thoroughly investigated in clinical studies and therapeutic outcomes for solid tumours.

**Methods:**

We collected numerous literatures to explain the efficacy of XPO1 Inhibitors in preclinical and clinical studies of a wide range of solid tumours.

**Results:**

In this review, we focus on the nuclear export function of XPO1 and results from clinical trials of its inhibitors in solid malignant tumours. We summarized the mechanism of action and therapeutic potential of XPO1 inhibitors, as well as adverse effects and response biomarkers.

**Conclusion:**

XPO1 inhibition has emerged as a promising therapeutic strategy in the fight against cancer, offering a novel approach to targeting tumorigenic processes and overcoming drug resistance. SINE compounds have demonstrated efficacy in a wide range of solid tumours, and ongoing research is focused on optimizing their use, identifying response biomarkers, and developing effective combination therapies.

**Key Points:**

- Exportin‐1 (XPO1) plays a critical role in mediating nucleocytoplasmic transport and cell cycle.
- XPO1 dysfunction promotes tumourigenesis and drug resistance within solid tumours.
- The therapeutic potential and ongoing researches on XPO1 inhibitors in the treatment of solid tumours.
- Additional researches are essential to address safety concerns and identify biomarkers for predicting patient response to XPO1 inhibitors.

## Graphical abstract

Exportin‐1 (XPO1), a key protein for nuclear export (proteins and RNAs) and cell cycle regulation, plays a significant role in the development of solid tumours and the emergence of drug resistance. While XPO1 inhibitors demonstrate promising therapeutic potential in preclinical and clinical studies for solid tumours, treatment safety and biomarkers of response require further exploration.

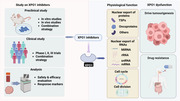

## 1 INTRODUCTION

The nuclear envelope separates eukaryotic cells into distinct nuclear and cytoplasmic compartments.^1^ The nuclear pore complex (NPC) serves as an essential conduit, facilitating the exchange of substantial quantities of protein and RNA cargoes between the nucleus and the cytoplasm, a critical process for maintaining optimal cellular function.^2^ Smaller molecules such as ions or metabolites can rapidly diffuse through the NPC, while larger entities such as proteins and RNA molecules are restricted and move less efficiently unless they assist a member of the karyopherin (Kap) β family of transport proteins.^3^ To date, more than 20 human Kaps have been identified. Although the functions of two isoforms of Kaps are unknown, 18 isoforms of Kaps are known to play crucial roles in nuclear transport, including import, export and biportin functions.^4^, ^5^ Kaps act as carrier molecules that facilitate cargo protein or RNA import into and export out of the nucleus via the NPC by recognising either a nuclear localisation signal (NLS) or a nuclear export signal (NES).^6^ Four classes of NLSs and one class of NES have been identified, including (i) the classic basic amino acid NLS sequences recognised by a heterodimer composed of importins α and β; (ii) the proline‐tyrosine NLS that binds KAPβ2 or KAPβ2b; (iii) the isoleucine‐lysine NLS that binds yeast Kap121; (iv) the transportin 3‐binding arginine‐serine repeat NLS found in RS domains; and (v) a hydrophobic leucine‐rich NES recognised by exportin‐1 (XPO1).^5^, ^6^

XPO1 plays an essential role as an exportin in transporting a diverse array of cargoes, including RNAs, tumour suppressor proteins and cell cycle regulators, such as p53, adenomatous polyposis coli (APC), retinoblastoma (RB), p21, p27, forkhead box protein O (FOXO), signal transducer and activator of transcription 3 (STAT3), inhibitor of nuclear factor kappa B (IκB) and breast cancer susceptibility gene 1 (BRCA1).^7^, ^8^, ^9^ XPO1 is overexpressed in numerous cancer tissues, and its overexpression is accompanied by disease progression, therapeutic resistance and decreased overall survival (OS) or progression‐free survival (PFS). Consequently, the potential of XPO1 as a therapeutic target for anticancer treatments has been widely researched over the past few decades.^7^, ^8^, ^9^

Selective inhibitor of nuclear export (SINE) compounds, which inhibit the expression of XPO1, represent a groundbreaking class of anticancer agents. Moreover, these agents are highly valued for their antitumour effectiveness in patients with various pretreated haematological malignancies and solid tumours.^10^, ^11^ Selinexor (an inhibitor of XPO1), the most prominent member of the SINE compound group, has been extensively evaluated in phase I and II clinical trials for various cancers. In addition, selinexor has achieved Food and Drug Administration(FDA) approval for the treatment of multiple myeloma (MM) and relapsed or refractory diffuse large B‐cell tumours.^12^, ^13^ Despite its primary application in treating haematologic malignancies, a plethora of preclinical studies and clinical trials suggest that selinexor also holds significant promise for the treatment of solid tumours. In this review, we provide a comprehensive introduction of the function of XPO1 and the role of XPO1 inhibitors in managing solid malignant tumours.

## 2 PHYSIOLOGICAL FUNCTIONS OF XPO1

*XPO1* was first isolated from *Schizosaccharomyces pombe* and was originally named chromosomal region maintenance 1 (*CRM1*); this gene maintains high‐order chromosomal structure.^14^ Subsequent studies revealed that CRM1 in *S. cerevisiae* mediates the nuclear export of proteins and mRNAs, and this gene was therefore renamed XPO1.^15^ As a member of the Kapβ family of transport factors, XPO1 has been identified as a primary nuclear export protein that accepts leucine‐rich NESs, which can be found in many proteins as well as RNAs, and then transports them to the cytoplasm via the support of the small guanosine triphosphate (GTPase) Ran.^16^, ^17^, ^18^, ^19^, ^20^ The concentration of the GTPase Ran determines the direction of nucleoplasmic transport. Ran guanine exchange factor, also called regulator of chromosome condensation 1 (RCC1), GTPase‐activating protein (RanGAP) and RanGTP‐binding protein 1/2 (RanBP1/2), plays important roles in maintaining the differential concentrations of Ran in the nucleus and cytoplasm. In the nucleus, RCC1 converts RanGDP to RanGTP, while in the cytoplasm, RanGAP and RanBP1/2 convert RanGTP to RanGDP.^21^ XPO1 binds cargo proteins/RNAs and RanGTP, assembling a stable ternary export complex that is collaboratively exported into the cytoplasm through the NPC. Once the complex is exported into the cytoplasm, it is disassembled via RanGAP, which promotes the dephosphorylation of RanGTP to RanGDP.^22^, ^23^ Interestingly, XPO1 is then recycled to the nucleus for subsequent transport.^24^

### 2.1 Structure of XPO1

**FIGURE 1:**
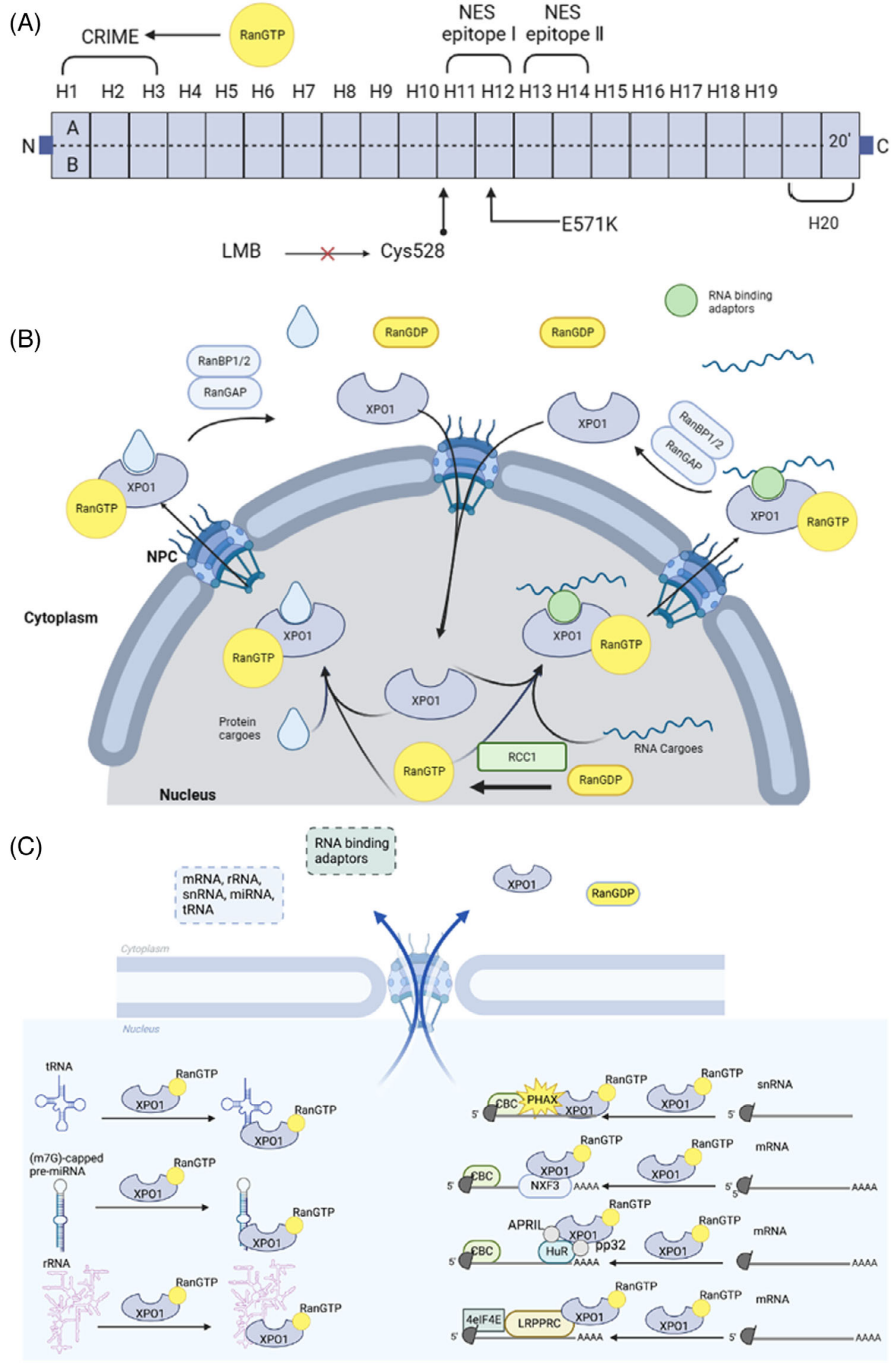
(A) Schematic structure of exportin‐1 (XPO1). Boxes 1−20 represent the HEAT repeat motifs. The CRIME domain (XPO1, importin β, etc.), which corresponds to HEAT repeats 1−3, shares homology with importin β and helps recognise RanGTP. H11‐12 and H13‐14 have two NES epitopes that can bind NES cargoes. However, Cys528 is modified by leptomycin B (LMB) and targets the central region of the XPO1 NES‐binding cleft. Moreover, a mutation hotspot (E571K) plays a role in oncogenic processes. (B) Schematic diagram of the physiological activities of XPO1. In terms of nuclear‐cytoplasmic transport, XPO1 and RanGDP undergo nuclear‐cytoplasmic transport via Ran guanine exchange factor (RanGEP), where they form XPO1‐RanGTP and further bind to cargo proteins. The substances involved in this translocation are generally proteins and RNAs, and the proteins can include tumour suppressors, cell cycle‐related proteins and oncogenes. When cargo is transported to the cytoplasm, the XPO1‐RanGTP complex and cargo are redissolved into XPO1, RanGDP and cargo (protein or RNA) with the help of GTPase‐activating protein (RanGAP) and RanGTP‐binding protein 1/2 (RanBP1/2). Among these components, XPO1 re‐enters the nucleus.^36^ (C) Schematic diagram of XPO1‐mediated RNAs nuclear export.

Native full‐length human XPO1 comprises 1071 amino acids and has 20 HEATs.^25^, ^26^ The canonical HEAT repeat is composed of two antiparallel helices, A and B, with approximately 40−50 residues, while H20, as a C‐terminal region, has three helices, H20', H20A and H20B.^25^ With the use of a combination of X‐ray crystallography, homology modelling and electron microscopy, the understanding of XPO1 function has improved.^27^ H1‐H3, the CRIME domain (CRM1, importin β, etc.), which shares homology with importin β, is an N‐terminal region that participates in RanGTP binding.^28^ Interestingly, NES cargos bind the outer surface of XPO1 in a bidirectional manner and interact with H11‐H12 and H13‐H14, two NES epitopes.^17^, ^25^, ^29^ XPO1 is known to recognise leucine‐rich NES proteins that bear a specific short peptide sequence comprising 10 ordered amino acid residues, defined as Φ1 × 2‐3Φ2 × 2‐3‐Φ3XΦ4. Here, Φ signifies isoleucine, leucine, methionine, phenylalanine or valine, and X represents any other amino acid.^30^ Interestingly, the location of XPO1 Cys528 within the leucine‐rich NES binding groove indicates its potential as a target for inhibiting the formation of export complexes.^31^ Leptomycin B (LMB) inhibits nuclear export by modifying the Cys528 residue of XPO1.^32^ A large‐scale analysis of 322 cancers revealed three hotspots of high‐recurrence mutations in *XPO1*, namely, E571, D624 and R749.^33^ E571K is the most common mutation of XPO1. The Q626 mutation of *XPO1* is second only to the E571 mutation in lung cancer, which is associated with poor prognosis.^34^ Moreover, the E571K mutation in *XPO1* has been identified as a key factor in cancer development because it augments the affinity between XPO1 and negatively charged C‐terminal NESs to promote nuclear export (Figure 1).^35^

### 2.2 Functions of XPO1

XPO1, the predominant protein of the Kap protein family, plays a critical role in shuttling numerous leucine‐rich NES proteins and RNAs from the nucleus, requiring the help of the small GTPases Ran and GTP to form a complex to promote importin‐RanGTP complex transfer to the cytoplasm.^20^ In addition to its significant role in nuclear‐cytoplasmic transport, XPO1 also plays an important role in the regulation of mitosis, specifically during the interphase of the cell cycle.^37^

#### 2.2.1 Protein export

XPO1 is essential for exporting NES proteins into the cytoplasm. Rev and the protein kinase inhibitor cyclic adenosine monophosphate (cAMP)‐dependent protein kinase were among the first proteins identified as NESs.^29^, ^38^ To date, the new database ValidNESs (http://prodata.swmed.edu/LRNes) shows that more than 221 proteins with diverse functions rely on XPO1 transport into the cytoplasm.^39^ These proteins, including cell cycle regulators (p21, cyclin B1, D1), tumour suppressor proteins (BRCA1/2, p53, APC, Rb, cellular‐abelsongene (c‐ABL)), antiapoptotic proteins (survivin) and autophagy‐related proteins (beclin‐1 and yes‐associated protein 1), are essential for regulating normal cellular physiological activities.^40^, ^41^, ^42^, ^43^, ^44^, ^45^ To date, extensive studies have been conducted on the proteins exported by XPO1. Further investigation of the cargo spectrum of XPO1 revealed additional functions involved in the spatial regulation of vesicle coat assembly, centrosomes, autophagy, peroxisome biogenesis, the cytoskeleton, ribosome maturation, translation and mRNA degradation, which suggests that protein export via XPO1 is a widespread and intricate process.^46^ The diverse functions of XPO1 indicate its potential as a therapeutic target in various diseases.

#### 2.2.2 RNA export

XPO1 was initially shown to export mRNA from the nucleus to the cytoplasm through its interaction with the RNA‐binding protein Rev.^38^ Subsequent evidence has demonstrated the vital function of XPO1 in the nuclear export of various types of RNA, such as ribosomal RNAs (rRNAs), uridine‐rich small nuclear RNAs (U snRNAs), specific mRNAs, miRNAs and transfer RNA (tRNAs).^18^, ^47^, ^48^ XPO1 is involved in the synthesis and processing of rRNA, in which it exports the pre‐40S and pre‐60S ribosomal subunits to the cytoplasm; this process is followed by splicing and translation.^49^, ^50^, ^51^, ^52^ An important aspect of the function of XPO1 is its regulation of heterogeneous nRNA (pre‐mRNA) splicing through its influence on the maturation of snRNAs.^53^ U snRNAs are transcribed in the nucleus and form complexes with phosphorylated adaptors for RNA export (PHAX), RanGTP, XPO1 and the cap‐binding complex. The complex is then transferred to the cytoplasm for further assembly and modification, where it eventually forms U snRNPs that re‐enter the nucleus for complete maturation and participation in splicing functions.^38^, ^54^, ^55^, ^56^, ^57^, ^58^ Although genome‐wide screens in *Drosophila* spp. have suggested that bulk mRNAs are exported through nuclear RNA export factor 1(NXF1)/TAP, which are expressed in all tissue types, subsequent research has demonstrated that XPO1 is involved in exporting a subset of specialised mRNAs in both humans and yeast.^59^, ^60^, ^61^ Additionally, research has revealed interactions between XPO1 and NXF3, a member of the NXF family, through a NES, which mediates the export of several mRNAs.^62^ XPO1 interacts with other adaptor proteins, including leucine‐rich pentatricopeptide repeat protein), eukaryotic translation initiator factor 4E (eIF4E) and HuR (with the help of APRIL and pp32), and facilitates the export of specific mRNAs.^63^ Moreover, XPO1 also plays a role in the export of several alternative small noncoding RNAs, including microRNAs and tRNAs.^61^ Most miRNAs are transcribed and possess cap structures as well as poly(A) tails to form primary miRNAs via RNA polymerase II in the nucleus.^64^ Long pri‐miRNAs are further processed into hairpin‐shaped precursor miRNAs (pre‐miRNAs) by a microprocessor that includes the double‐stranded RNase III enzyme DROSHA and its necessary cofactors, double‐stranded RNA‐binding protein DiGeorge syndrome critical region 8.^65^, ^66^, ^67^ Previous studies have shown that XPO5 mediates the nuclear transport of pre‐miRNAs.^68^, ^69^ Subsequent studies have demonstrated that XPO1 selectively transports a novel class of (m7G)‐capped pre‐miRNAs.^70^, ^71^

#### 2.2.3 Other functions of XPO1

An increasing body of evidence has demonstrated the critical role of XPO1 not only in mediating nucleocytoplasmic transport, cell division and the cell cycle but also in spindle formation.^72^ XPO1, located on kinetochores, has been found to stabilise microtubule movement and support chromosome segregation by forming a complex with RanGAP1 and RanBP2.^73^, ^74^, ^75^ During the cell cycle, XPO1 is present at the centrosome, where it assists in the recruitment of pericentriolar proteins as well as in spindle localisation.^76^ Abnormal expression of XPO1 is linked to cellular instability. Elevated XPO1 expression potentially accelerates the cell cycle, while decreased or absent XPO1 expression might disrupt the integrity of mitogenic granules and telomeres.^77^, ^78^ Furthermore, XPO1 may perform a regulatory function in early embryonic development, highlighting its broad influence on cellular processes.^79^

## 3 ROLES OF XPO1 IN CANCER

**FIGURE 2:**
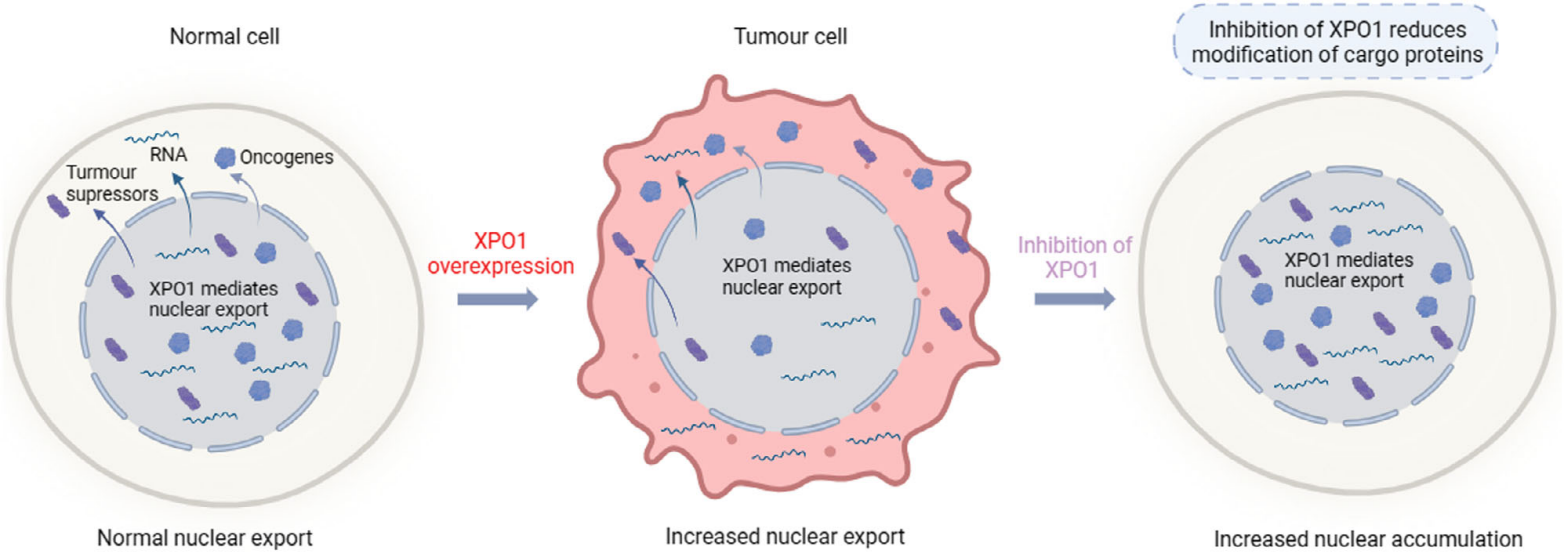
The mechanism of XPO1 in normal and malignant cells. In normal cells, XPO1 maintains normal nuclear export, while in tumour cells, increased XPO1 expression promotes nuclear export leading to a large number of mislocated proteins and RNAs in the cytoplasm. Inhibition of XPO1 induced nuclear accumulation of tumour suppressors, oncogenes and RNAs.

XPO1 is a critical nuclear export protein that plays a vital role in sustaining normal cellular physiological functions.^80^ Intriguingly, XPO1 expression is markedly increased in a wide array of tumour cells, including osteosarcoma, glioma, lung cancer, ovarian cancer, pancreatic cancer, oesophageal cancer, gastric cancer and colorectal cancer (CRC) cells. The upregulation of XPO1 expression disrupts the balance of nucleoplasmic transport and leads to the mislocalisation of tumour suppressor proteins and oncoproteins (Table 1), which consequently promotes tumour progression and potentially fosters therapeutic resistance.^81^, ^82^, ^83^, ^84^, ^85^, ^86^, ^87^, ^88^ Despite its recognised significance, the mechanism underlying the overexpression of XPO1 in numerous tumours has not been elucidated. Accumulating evidence suggests a correlation between the aberrant nucleocytoplasmic transport and elevated XPO1 expression in tumours, leading to the occurrence and development of several cancers.^22^ The upregulation of XPO1 allows key cellular proteins, such as p27Kip1 and p53, to be transported into the cytoplasm and inactivates inappropriate cell growth or DNA repair.^89^ Similarly, oncogenes such as STAT3 and c‐myc activate downstream signalling pathways to stimulate proliferation and inhibit apoptosis upon export.^89^ Inhibition of XPO1 promotes the nuclear accumulation of TSPs and oncogenes and promotes the apoptosis of tumour cells.^90^ Several studies have shown that inhibiting XPO1 induces tumour cell apoptosis by triggering intranuclear accumulation of p53 or p27.^91^, ^92^ STAT3 phosphorylation or acetylation activates downstream growth signals, promotes cell proliferation and prevents apoptosis.^93^ The CREB‐binding protein (CBP) induces acetylation of STAT3 to regulate transcriptional activity.^94^ XPO1 inhibition represses STAT3 activation by affecting CBP activity to reduce the level of STAT3 acetylation, which blocks the interaction between STAT3 and the survivin promoter.^95^ C‐myc plays a key role as a regulator in a variety of tumours.^96^ mTOR‐dependent 4EBP1 phosphorylation has been shown to be a key factor by which myc maintains tumour survival.^97^ eIF4E, a translation regulator downstream of mTOR, plays an important role in myc transcriptional regulation.^98^ KPT‐330 inhibits the nuclear export of c‐myc mRNA by inducing the nuclear accumulation of XPO1 and eIF4E complexes.^99^ Subsequent studies have shown that inhibition of XPO1 also regulates c‐myc expression through other pathways, such as the upregulation of miR‐145.^100^ Therefore, inhibition of XPO1 may constitute a new strategy for tumour therapy (Figure 2).

**TABLE 1:** Exportin‐1 (XPO1) mediates the nuclear export of cancer‐related cargoes.

Category	Cargo	Biological process	Related cancer
Tumour suppressor proteins	APC	Wnt signaling pathway	Colorectal cancer (CRC)^261^
	p21 Cip1	Cell cycle	Breast cancer, chronic myeloid leukaemia
	p53	Apoptosis, cell cycle, DNA damage, DNA repair	Ovarian cancer, breast cancer, lung cancer, gastric cancer, gallbladder cancer, neuroblastoma, CRC^8^, ^111^, ^201^, ^262^, ^263^, ^264^, ^265^
	p27 Kip1	Cell cycle	Cholangiocarcinoma, osteosarcoma^266^, ^267^
	RB	Transcription factor	Lung cancer, cervical cancer^90^, ^268^
	PALB2	DNA repair	Breast cancer, pancreatic cancer^269^, ^270^
	TET2	Cell cycle	Small intestinal neuroendocrine tumours^271^
	FOXO	Apoptosis, transcription regulation	Breast, prostate, thyroid cancer, glioblastoma, melanoma^114^, ^272^
	VDUP1	Tumour and metastasis suppressor	Gastric cancer, cervical cancer, lung cancer^273^
	MLH1	Cell cycle, DNA damage, DNA repair	CRCs^274^
	KLF6	Transcription regulation	Prostate cancer, gastric cancer^275^, ^276^
	RASSF2	Cell growth	Lung cancer^277^
	TOB	TOB/BTG antiproliferative (APRO) protein family	Breast cancer^278^
Oncoproteins	Cyclin D1	Cell cycle, cell division, DNA damage	Prostate cancer, multiple myeloma (MM)^279^, ^280^
	FAM21	Protein transport	Pancreatic cancer^281^
	AKT1	Apoptosis, carbohydrate metabolism	Thyroid cancer, breast cancer^133^, ^282^
	SMAD4	Transcription regulation	Pancreatic cancer, CRC^283^, ^284^
Anti‐apoptotic proteins	Nucleophosmin	DNA repair, cell cycle	Acute myeloid, leukemia^285^
	Survivin	Apoptosis, cell cycle	Breast cancer, liposarcoma, prostate cancer, ovarian cancer^95^, ^286^, ^287^
Transcription factors	RUNX	Transcription factor	Breast cancer, gastric cancer, skin squamous cell carcinoma, esophageal cancer, lung cancer, colon cancer, pancreatic cancer, prostate cancer^288^, ^289^
	IκBα	Transcription factor	MM, lung cancer, breast cancer, chronic lymphocytic leukemia, T‐cell lymphoma, ovarian cancer^192^, ^290^, ^291^, ^292^, ^293^, ^294^

Abbreviations: APC, adenomatous polyposis coli; FOXO, forkhead box protein O; IκBα, inhibitor of nuclear factor kappa B alpha.

## 4 ROLES OF XPO1 IN DRUG RESISTANCE

**FIGURE 3:**
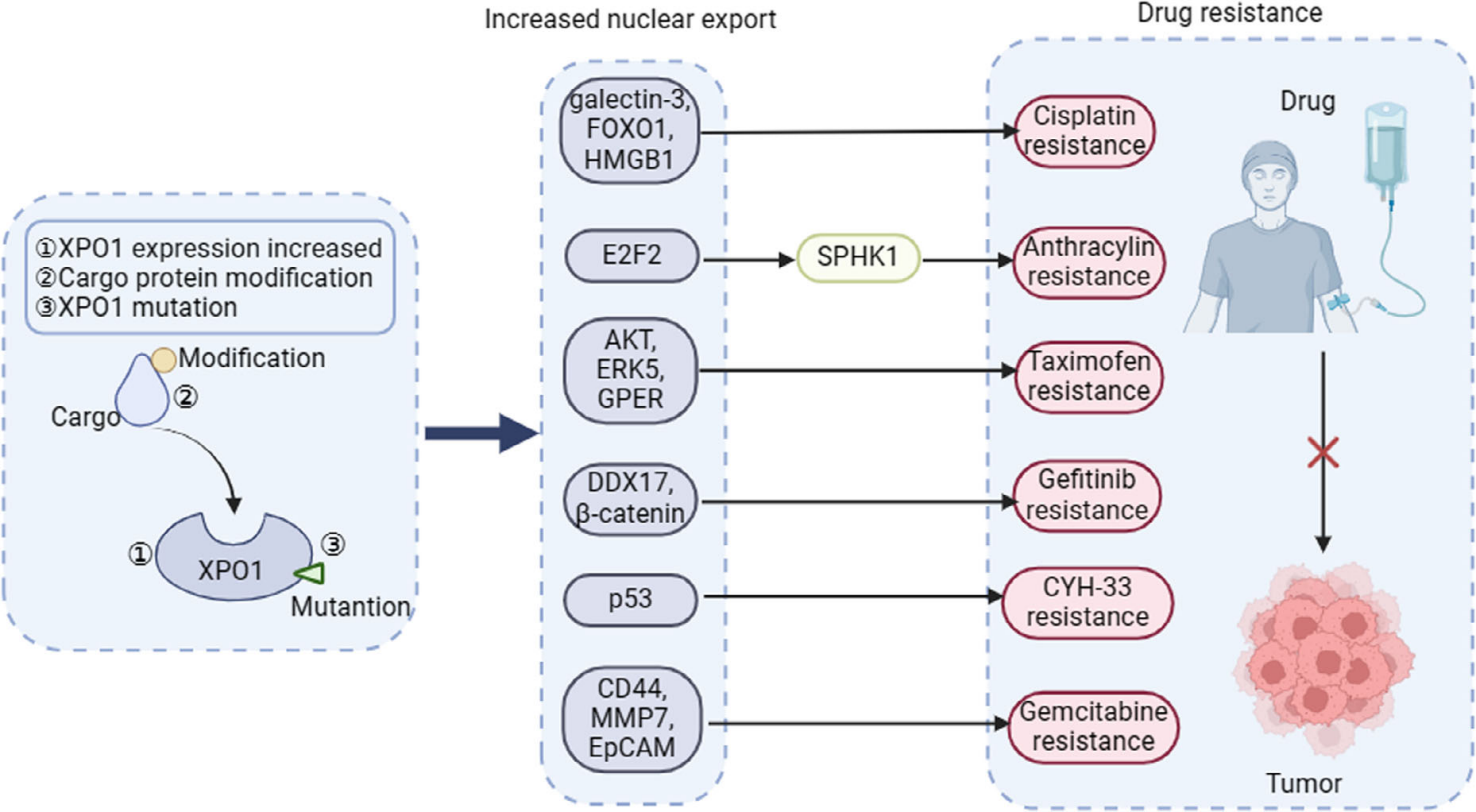
Roles of XPO1 in drug resistance. Cargo protein modification, XPO1 mutation or increased XPO1 expression promote nuclear export and induce tumour resistance.

Drug resistance is an inevitable problem in cancer treatment. Accumulating evidence suggests that XPO1 may mediate drug resistance in multiple tumour types by promoting the nuclear export of multiple drug targets. Protein mislocalisation via XPO1 is a significant contributor to tumour drug resistance.^101^ We classified the following as several reasons for increased nucleoplasmic transport (Figure 3). High XPO1 expression often indicates poor survival and chemical resistance.^102^ In breast cancer patients >40 years of age, cytoplasmic BRCA1 expression was inversely associated with metastasis‐free survival.^103^ Inhibition of XPO1 sensitised hepatocellular carcinoma cells to sorafenib.^104^ NESs on cargo proteins can be exposed through different modifications, including phosphorylation, dephosphorylation, acetylation, SUMOylation and ubiquitination, which induce export.^105^ Abnormal activation of signalling pathways plays an important role in NES exposure. Overactivation of protein kinase B (AKT) induces FOXO phosphorylation and promotes XPO1‐mediated FOXO nucleoplasmic transport, which causes FOXO to lose its ability to induce apoptosis and transcriptional regulation.^106^ Notably, the *XPO1* E571K mutation was shown to have greater affinity for negatively charged C‐terminal NESs than for other NESs, which may lead to the mislocalisation of several proteins. The *XPO1* E571K mutation promotes the accumulation of TRAF2 (tumour necrosis factor receptor‐associated factor 2) in the cytoplasm, which is negatively correlated with the survival of CRC patients.^33^, ^107^

### 4.1 Platinum‐based chemotherapeutic agents

Moreover, platinum resistance frequently impedes the effectiveness of chemotherapy, and numerous factors contribute to this resistance. Several studies have shown that increased expression of galectin‐3 (Gal‐3) decreases sensitivity to platinum chemotherapy drugs.^108^, ^109^ Gal‐3, a member of the β‐galactoside‐carbohydrate binding protein family, is associated with carcinogenesis and malignant potential in the majority of tumours.^110^ In response to apoptotic stimuli, Gal‐3 translocates from the nucleus to the cytoplasm to prevent apoptosis. It has been reported that phosphorylated Gal‐3 at serine 6 enhances nuclear export and promotes antiapoptotic activity. Another study revealed that XPO1 expression was increased in cisplatin‐resistant ovarian cancer cells.^111^ Breast cancer tumour cells exposed to LMB or cisplatin retain Gal‐3 in the nucleus, where it induces apoptosis.^108^ KPT‐185 and KPT‐330 resensitise cisplatin‐resistant ovarian cancer cells to cisplatin by inhibiting XPO1 expression.^111^ Thus, inhibition of XPO1 maintains the nuclear localisation of Gal‐3, inducing apoptosis in platinum‐resistant cells.^101^, ^112^ Subsequent studies revealed that FOXO1 and high mobility group box 1 (HMGB1), which participate in DNA repair and extracellular signalling, play important roles in platinum resistance.^113^, ^114^ One study showed that increased FOXO1 nuclear translocation decreased cisplatin sensitivity in non‐small cell lung cancer (NSCLC).^115^ Similarly, cisplatin induces HMGB1 expression in tumour cells, which in turn suppresses cisplatin‐induced apoptosis.^116^ The suppression of XPO1 expression by KPT‐330 triggers the nuclear relocation of FOXO1 and HMGB1, thereby overcoming platinum resistance in tumour cells.^113^, ^114^

### 4.2 Anthracycline drugs

Despite medical advancements, the mortality rate of head and neck squamous cell carcinoma (HNSCC) has remained consistent for several decades at approximately 45%.^117^ This persistent statistic is largely attributable to the development of drug resistance in patients. Several studies have revealed that in more than 80% of patients with HNSCC, the transcription factor E2F7 is mislocalised to the cytoplasm.^118^, ^119^ E2F7 regulates the production of proteins that confer resistance to a class of chemotherapy drugs known as anthracyclines. One study showed that E2F7 expression was positively correlated with sphingosine kinase 1 (SPHK1) expression.^117^ SPHK1 and SPHK2 (two sphingosine kinase isoforms) catalyse the adenosine triphosphate (ATP)‐dependent phosphorylation of sphingosine to form sphingosine 1‐phosphate (S1P), which is responsible for the regulation of a wide range of biological effects, including proliferation and survival.^120^ The phorbol 12‐myristate 13‐acetate (PMA, a protein kinase C activator)‐induced translocation of SPHK1 to the plasma membrane significantly increased SPHK1 activity.^121^ Several studies have suggested that anthracycline resistance in osteosarcoma may be related to increased expression of SPHK1.^122^, ^123^ Phenoxodiol blocks the activation of SPHK1, and anthracycline is then able to promote ceramide accumulation in osteosarcoma cells, mediate the activation of the apoptosis signal‐regulating kinase 1 (ASK1)/c‐Jun N‐terminal kinase (JNK) pathway and negatively regulate Akt activity, suggesting that the inhibition of SPHK1 may restore the sensitivity of osteosarcoma to anthracycline.^122^ Similarly, E2F7‐induced doxorubicin resistance is mediated by SPHK1‐dependent AKT activation in squamous cell carcinomas.^117^ Furthermore, KPT‐330 has been shown to mediate the nuclear retention of E2F7, leading to the repression of SPHK1, which in turn increases anthracycline sensitivity^119^ These findings reveal some of the mechanisms underlying anthracycline resistance and provide new treatment strategies that warrant further clinical evaluation in patients with HNSCC and osteosarcoma.

### 4.3 Tamoxifen

Tamoxifen is one of the most potent endocrine therapeutic agents for treating estrogen receptor (ER)α [+] in breast cancer. However, the effectiveness of endocrine therapies has limitations, as evidenced by instances of tumour recurrence or metastasis in ERα (+) patients.^124^, ^125^ Tamoxifen resistance involves complex mechanisms, including loss of ER, increase in growth factor receptors (such as human epidermal growth factor receptor 2HER2) and aberrant activation of signalling pathways (such as phosphoinositide 3‐kinase (PI3K)/AKT signalling).^126^ Recently, the protein interferon alpha inducible protein 27 (IFI27) was shown to reduce tumour cell sensitivity to tamoxifen by mediating the nuclear export of ERα via XPO1.^124^ HER2 overexpression was associated with poor prognosis in patients treated with tamoxifen, mainly due to the activation of other survival signalling pathways (such as the PI3K/AKT signalling pathway).^127^ The expression of XPO1 and HER‐2 is positively correlated, and inhibition of XPO1 induces HER‐2 nuclear accumulation.^87^, ^128^ The G‐protein‐coupled ER (GPER) is strongly associated with tamoxifen resistance in breast cancer.^129^ A subsequent study revealed that tumour cells induced GPER nuclear export via an XPO1‐dependent pathway in cancer‐associated fibroblasts by activating the PI3K/AKT signalling pathway.^130^ Several studies have shown that inhibiting the activation of the PI3K/AKT signalling pathway increases the sensitivity of breast cancer to tamoxifen.^131^, ^132^ In addition, tamoxifen treatment induced an increase in XPO1 expression, which may suggest that the subcellular localisation of key proteins is related to the tamoxifen treatment response.^125^ One study showed that KPT‐330 enhanced the sensitivity of breast cancer cells to tamoxifen by inhibiting the nuclear export of ERK5.^125^ However, there is a risk of tumour recurrence in patients treated with KPT‐330 alone, which warrants further exploration of the combination of KPT‐330 and tamoxifen. Interestingly, the combination of XPO1 and tamoxifen blocked the activation of the AKT signalling pathway, induced autophagy, improved the treatment sensitivity to tamoxifen and reshaped the tumour metabolic pathway, which provided a new approach for the future treatment of breast cancer.^133^, ^134^

### 4.4 Gefitinib

Lung cancer remains the leading cause of cancer‐related deaths worldwide.^135^ NSCLC represents approximately 85% of all lung cancer cases. Currently, epidermal growth factor (EGF) receptor tyrosine kinase inhibitors (EGFR‐TKIs), such as gefitinib, are the primary treatment for advanced NSCLC patients with EGFR mutations.^136^ Despite the initial efficacy of EGFR‐TKIs, approximately 65% of EGFR‐TKI‐sensitive NSCLC patients develop resistance to these medications within 9 to 13 months of treatment.^137^ DEAD‐box helicase 17 (DDX17) is a multifunctional ATP‐dependent RNA/DNA helicase that is involved in the initiation and development of a variety of tumours.^138^ In NSCLC, the upregulation of DDX17 is associated with increased gefitinib resistance, while the silencing of DDX17 can partially reverse this sensitivity.^139^ One study showed that increased DDX17 expression promoted β‐catenin transcriptional activity.^140^ As a member of the canonical Wnt pathway, β‐catenin participates in the regulation of cell proliferation through nuclear translocation.^141^ Several studies have shown that gefitinib resistance is associated with increased β‐catenin transcriptional activity and that inhibition of β‐catenin partially restores gefitinib sensitivity.^142^, ^143^ Furthermore, blocking DDX17 nuclear export by inhibiting the binding of XPO1 significantly reduced the nuclear translocation of β‐catenin, which improved the sensitivity of NSCLC to gefitinib.^139^ These results provide a new therapeutic strategy for the clinical treatment of gefitinib‐resistant NSCLC.

### 4.5 Other drugs

Gemcitabine is a basic chemotherapy drug that is often limited by patient tolerance.^144^ Gemcitabine resistance is extremely complex and involves alterations in the tumour microenvironment.^145^ One study revealed that the AKT, p53 and hypoxia‐inducible factor‐1 signalling pathways are associated with gemcitabine resistance.^145^ The key molecules of the above pathways are regulated by the nuclear export of XPO1. Thus, inhibition of nuclear export appears to ameliorate gemcitabine resistance. A previous study showed that KPT‐330 enhanced the antitumour effect of gemcitabine by inducing the nuclear accumulation of p27 and decreasing survivin.^146^ Recently, a study revealed that combination treatment with KPT‐330 and gemina‐nab‐paclitaxel improved the survival time of Pdx‐1‐Cre (KPC) model mice.^147^ CD44, a cell surface protein, has been reported to be associated with gemcitabine resistance.^148^ The combination of KPT‐330 and gemcitabine‐nab‐paclitaxel significantly reduced the number of CD44‐positive cells. The extracellular matrix between tumour cells hinders contact between gemcitabine and tumour cells, leading to the development of gemcitabine resistance.^149^ The combination of KPT‐330 and gemcitabine‐nab‐paclitaxel decreased MMP7 and epithelial cellular adhesion molecule, which play important roles in the regulation of the ECM, and increased the therapeutic sensitivity to gemcitabine.^147^

Similarly, XPO1 also mediates resistance to other drugs. The application of KPT‐330 to CYH33‐resistant breast cancer cells enhances the localisation of p53 in the nucleus, leading to cell cycle arrest and a reversal of CYH33 resistance.^150^ Lenvatinib resistance is a common clinical hurdle in the treatment of thyroid cancer. Research has indicated that an XPO1 inhibitor can suppress the growth of lenvatinib‐resistant 8505C thyroid mesenchymal carcinoma cells both in vivo and in vitro.^151^

## 5 PAST, PRESENT,AND FUTURE OF *XPO1* INHIBITORS

### 5.1 XPO1 inhibitors in *preclinical and* early clinical *development*

XPO1 has emerged as a promising potential target in cancer therapy because of its consistent involvement in a wide range of tumour types. Nevertheless, the development of XPO1 inhibitors has encountered substantial challenges. Natural inhibitors of XPO1 (Table 2) and its derivatives have been linked to severe toxic side effects, prompting researchers to explore the potential of small molecule inhibitors.^152^, ^153^, ^154^, ^155^, ^156^ There are many ongoing studies regarding SINE compounds. Notably, KPT‐330 has achieved FDA approval for the treatment of MM and relapsed or refractory diffuse large B‐cell tumours, marking a significant step forward in the field.^12^, ^13^

**TABLE 2:** Natural products of XPO1 inhibitor.

XPO1 inhibitor drugs	Function	Restriction	Effect in clinical trial
LMB	Alkylated amino acid residues of Cys528 at the active site of XPO1^295^, ^296^, ^297^, ^298^, ^299^, ^300^	Severe malaise and anorexia^301^	No partial response
Anguinomycins		NR	NR
Ratjadones		High cytotoxicity^302^	NR
Piperlongumine		NR	Effective inhibition of human acute myeloid leukaemia cells^303^
Curcumin		Low oral bioavailability^304^	>120 clinical trials, but no validated clinical trials have been successful^304^, ^305^
Plumbagin		Limited solubility, oral bioavailability and unknown toxicity^306^	NR
Oridonin	Increased the expression of nucleoporin98^307^	Uncertain toxicity^308^	NR

Abbreviations: LMB, Leptomycin B; NR, not reported.

### 5.2 Natural inhibitors and their analogues

LMB is an antifungal agent generated by the bacterium *Streptomyces* that is an inaugural natural inhibitor capable of inhibiting XPO1 to achieve antitumour effects.^154^, ^157^, ^158^ Regrettably, the corresponding clinical study abruptly ended due to the intense severity of the toxic side effects of LMB, which included severe malaise and anorexia and no partial response (PR).^159^ Subsequent discoveries of natural inhibitors akin to LMB, including anguinomycins and ratjadones A through D, presented significant challenges for use in oncological treatment, primarily due to their debilitating toxicity similar to that of LMB.^160^, ^161^

Several natural XPO1 inhibitors, including piperlongumine, curcumin, plumbagin and oridonin, have been identified in plant extracts. These compounds have demonstrated potential antitumour effects across a variety of tumour cells.^162^, ^163^, ^164^, ^165^, ^166^, ^167^, ^168^, ^169^, ^170^, ^171^, ^172^, ^173^ Similarly, semisynthetic inhibitors exhibit significant antitumour efficacy. However, the advancement of these semisynthetic inhibitors in clinical studies has been impeded due to potential toxicological responses that remain unverified.^174^, ^175^

### 5.3 Selective inhibitors

**FIGURE 4:**
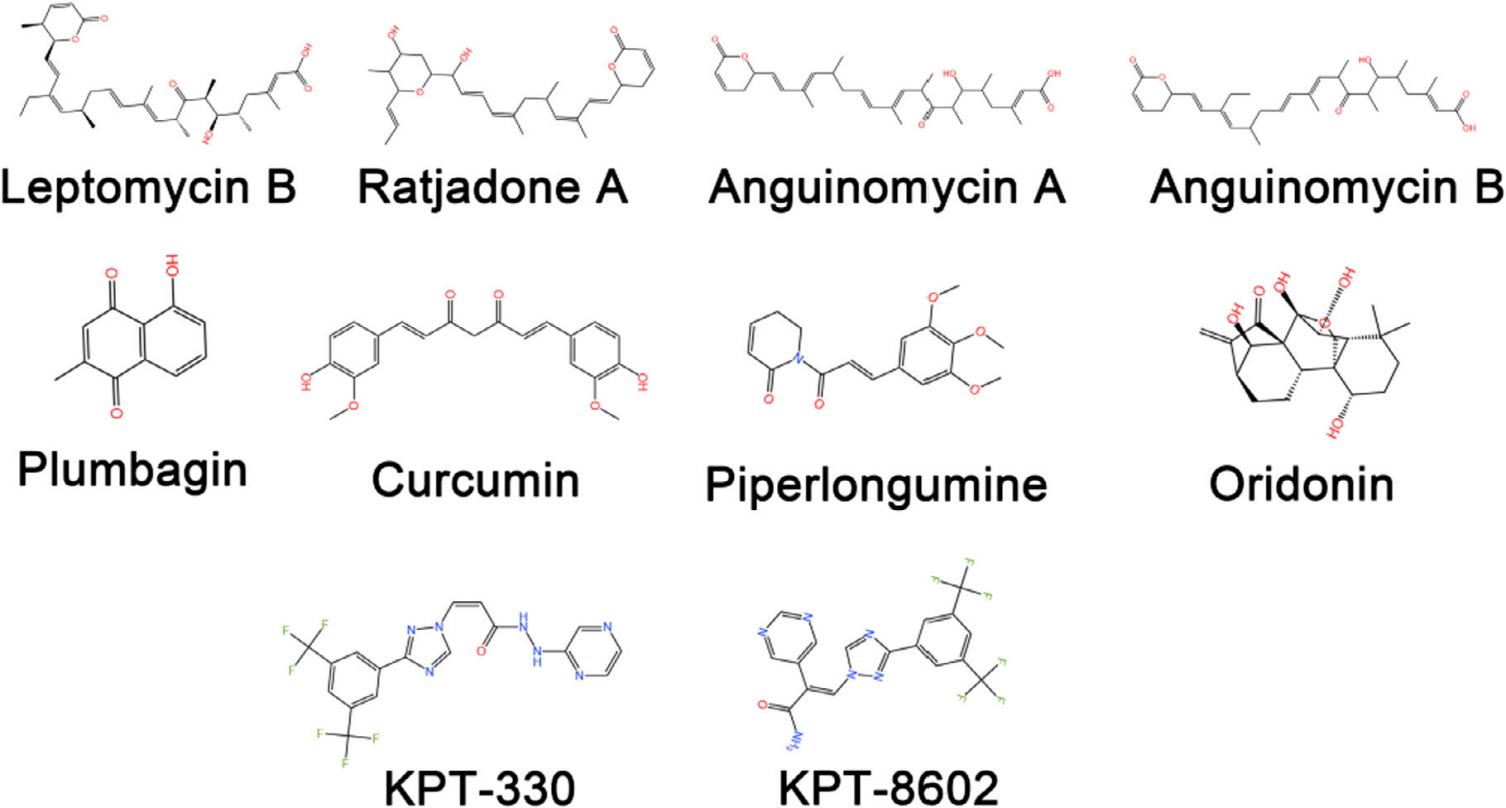
Structures of XPO1 inhibitors.

To mitigate the toxic side effects associated with LMB and its analogues during clinical trials, researchers have used the consensus‐induced fit docking methodology. This approach not only decreases the financial burden of drug development but also enhances the diversity of potential therapeutic agents. Utilising computer simulations within dedicated research is particularly effective for creating compounds that are collectively known as SINE compounds (Figure 4).^176^

The SINE compounds include KPT‐301, KPT‐330, KPT‐335, KPT‐185, KPT‐276, KPT‐251 and KPT‐8602. KPT‐301, a trans isomer of KPT185, is often used as a negative control for SINEs due to its low activity.^177^, ^178^ Extensive research has indicated that KPT‐185 has robust antitumour effects in vitro. However, the efficacy of the antitumour effects of KPT‐185 in vivo did not meet the anticipated expectations. Subsequent clinical trials have shown encouraging antitumour outcomes. Remarkably, KPT‐330 gained the approval of the FDA for treating MM and relapsed or refractory diffuse large B‐cell tumours.^12^, ^13^, ^179^, ^180^, ^181^ Collectively, these findings underscore the therapeutic potential of SINE compounds in the management of tumours.

## 6 THE STUDY OF SINE COMPOUNDS IN VARIOUS SOLID TUMOURS

Following the remarkable success of KPT‐330 in treating haematological malignancies, a crucial question arises: Do KPT‐330 and its analogous derivatives demonstrate significant antitumour efficacy in solid tumours?

### 6.1 Glioblastoma

KPT‐330 and KPT‐276 effectively inhibited the growth of glioma cells in vitro and enhanced survival rates in an orthotopic patient‐derived xenograft (PDX) model.^182^, ^183^ KPT‐330 treatment exhibited a potent proapoptotic effect on tumour cells, and its efficacy was notably augmented when KPT‐330 was combined with Bcl‐xL and the Bcl‐2 inhibitor ABT263, compared to that of the individual treatments, thereby leading to superior tumour suppression both in vivo and in vitro.^184^, ^185^ Intriguingly, while treatment with KPT‐330 or the XPO1 inhibitor S109 alone did not markedly improve survival rates in vivo, it amplified the sensitivity of tumour cells to radiotherapy, hence offering a fresh perspective for future treatment strategies.^182^, ^186^ Moreover, studies on high‐grade gliomas have shown that nerve growth factor receptors heighten the responsiveness of KPT‐330, a discovery that could mitigate the issue of single‐drug resistance and provide insights for future combination therapies.^187^

### 6.2 Lung cancer

KPT‐330 inhibited the proliferation of 11 NSCLC cell lines in vitro, and combination with either cisplatin or the Bcl‐xL inhibitor A‐1331852 resulted in more pronounced inhibition than a single treatment.^184^, ^188^ XPO1 treatment in combination with cisplatin/irinotecan or in combination with the poly (adenosine diphosphate (ADP)‐ribose) polymerase (PARP) inhibitor BMN673 has significant efficacy in vivo, ameliorating chemoresistance.^189^, ^190^ In addition, XPO1 was surprisingly effective in treating kirsten ratsarcoma viral oncogene homolog (KRAS) ‐mutant lung cancer; in combination with sotorasib (a KRAS‐mutant inhibitor), XPO1 not only inhibited tumour cell proliferation in vitro but also effectively inhibited *KRAS12C*‐mutated lung cancer cells in SCLC xenografts, increasing the chances of survival in mice.^191^, ^192^, ^193^ Although *XPO1* has an extremely low mutation rate, the occurrence of a mutation poses a significant survival risk for patients with NSCLC, and further research is needed.^34^

### 6.3 Breast cancer

KPT‐185, KPT‐251, KPT‐276 and KPT‐330 have been shown to significantly inhibit the growth of breast cancer cell lines both in vivo and in vitro.^95^, ^194^ Research has indicated that KPT‐330 combined with paclitaxel or eribulin has greater antitumour efficacy than carboplatin or doxorubicin alone, both in vitro and in triple‐negative breast cancer (TNBC) PDX models.^195^, ^196^ In a separate study, KPT‐330, when used in combination with GSK2126458, a PI3K/mTOR inhibitor, was shown to significantly reduce tumour burden, compared to monotherapy in TNBC PDX models.^197^ Moreover, the binding of KPT‐330 to the TRAIL‐R2xCD3 BsAb has been shown to stimulate apoptosis in TNBC cells.^198^ KPT‐330 combined with olaparib enhances antitumour activity in vivo and in vitro, regardless of the presence of a *BRCA1* mutation in TNBC, indicating a potential direction for future treatment strategies.^199^ It has also been suggested that the antitumour and antiproliferative effects of KPT‐330 can be amplified by elevating miR‐34a levels, enhancing the effectiveness of treatment for TNBC.^200^ The combination of KPT‐330 and tamoxifen has been shown to be effective in treating tumours in tamoxifen‐resistant tumour xenografts and in vitro. These findings suggest that KPT‐330 could restore the sensitivity of tamoxifen‐resistant cells and prevent recurrence in vivo.^125^, ^133^, ^134^ Additionally, for *TP53* wild‐type (WT) breast cancer, KPT‐330 may enhance proliferation inhibition when combined with tucidinostat, a type I histone deacetylase inhibitor, in vitro and significantly inhibit tumour growth in cell‐derived xenografts (CDXs).^201^

### 6.4 Pancreatic cancer

KPT‐185 or KPT‐330, when used individually, effectively inhibited the proliferation of pancreatic cancer cells and demonstrated significant efficacy in halting tumour growth in xenograft models.^100^, ^146^, ^202^ KPT‐330 treatment in combination with gemcitabine markedly enhances the antitumour effects of KPT‐330 against pancreatic cancer both in vitro and in vivo, leading to a decrease in tumour volume and a reduction in liver metastasis.^146^ In addition, another study revealed that combining KPT‐330 with gemcitabine supplemented with nab‐paclitaxel resulted in synergistic inhibition of pancreatic cancer cell proliferation. This combination was more effective at halting the growth of PDXs and orthotopic tumours than was any monotherapy.^203^ Among pancreatic cancers characterised by high rates of *KRAS* mutation, KPT‐330 has been found to counteract the drug resistance of KRAS G12C‐resistant pancreatic cancer cells. Combining KPT‐330 with KRAS G12C inhibitors not only drastically inhibited the proliferative capacity of these drug‐resistant cell lines in vitro but also exerted significant tumour‐suppressing effects in a CDX model.^191^

### 6.5 CRC

KPT‐330 effectively impeded the proliferation of CRC cell lines in vitro, and its synergistic use with proteasome inhibitors significantly enhanced its antitumour effect both in vitro and in vivo.^9^, ^204^ Furthermore, a subsequent study indicated that a sequential regimen involving KPT‐330/KPT‐8602 and the ataxia telangiectasia and Rad3‐related (ATR) inhibitor AZD6738 offered superior efficacy, compared to either agent used independently, thereby extending survival time in *TP53*‐mutant models of CRC.^205^ KPT‐330 also amplified tumour radiosensitivity, thereby bolstering tumour suppression in vitro and in vivo.^206^, ^207^ These investigations could lead to valuable reference points for devising future strategies for combination therapy involving the use of other drugs for tumour management.

### 6.6 Cervical cancer

The administration of LMB to HeLa cells suppressed cellular resistance in vitro and additionally hindered the recovery of apoptotic cells.^208^ In a separate study, the combination of KPT‐330 and A‐1331852 was found to effectively stimulate apoptosis in HeLa cells in a controlled laboratory environment.^184^

### 6.7 Ovarian cancer

Research has indicated that KPT‐330 and KPT‐185 have a profound impact on cell apoptosis in vitro, markedly increasing the percentage of apoptotic cells. These agents have also been demonstrated to significantly inhibit tumour proliferation in human ovarian carcinoma xenografts and orthotopic models.^114^, ^196^, ^209^, ^210^ KPT‐330 in combination with cisplatin exhibited an enhanced tumour‐suppressing effect on platinum‐sensitive ovarian cancer cells. These synergistic effects work by boosting platinum sensitivity in these cancer cells.^111^, ^114^ In addition, KPT‐330 combined with other drugs, such as RG7388 (an MNM2 inhibitor) or olaparib, synergistically impedes ovarian cancer cell proliferation in vitro by promoting apoptosis. The combination of KPT‐330 with topotecan or olaparib significantly decreases tumour nodule size, inhibits tumour cell proliferation and extends survival in vivo.^196^, ^209^, ^210^ To address the issue of tumour cell resistance caused by the long‐term use of SINEs, strategies such as inhibiting the NRG1/EBB3 signalling pathway have shown potential effectiveness.^211^

### 6.8 Prostate cancer

Various SINEs, such as KPT‐127, KPT‐185, KPT‐205, KPT‐225, KPT‐251 and KPT‐330, have been found to inhibit the growth of prostate cancer cells by inducing apoptosis and arresting the cell cycle in vitro.^40^, ^177^, ^212^ However, the inhibitory effect of KPT‐301 on prostate cancer cells was not obvious in vitro.^177^ Another study showed that KPT‐330 or KPT‐251 in a systemic metastasis model significantly reduced the likelihood of bone metastasis from prostate cancer and improved survival time in mice.^212^ Interestingly, KPT‐330 or KPT‐251 not only enhanced the effectiveness of the chemotherapeutic drug docetaxel (DTX) in DTX‐sensitive prostate cancer cells but also resensitised DTX‐resistant prostate cancer cells. When these agents are combined with DTX, tumour cell proliferation is inhibited, and apoptosis is induced, effectively extending the survival time of mice.^213^ To improve the effectiveness of treatment for desmoplastic‐resistant prostate cancer, KPT‐330 was combined with anti‐androgen receptor (AR) drugs, such as enzalutamide, which have been shown to synergistically enhance antitumour effects in vitro and vivo. Similarly, KPT‐8602, which is used in combination with these anti‐AR agents, has been shown to synergistically inhibit tumour growth and prolong the survival time of mice in vivo.^214^ Furthermore, the pairing of KPT‐8602 with PARP inhibitors demonstrated increased effectiveness in inhibiting the proliferation of castration‐resistant prostate cancer cells by inducing apoptosis.^215^

### 6.9 Sarcoma

Extensive research has illuminated the notable tumour‐suppressing potential of SINEs in different types of sarcomas, including gastrointestinal mesenchymal tumours (GISTs), liposarcomas, smooth muscle sarcomas, rhabdomyosarcomas, undifferentiated sarcomas, alveolar soft part sarcomas (ASPSs), Kaposi's sarcomas, Ewing sarcomas (EWSs) and osteosarcomas.^216^, ^217^, ^218^, ^219^, ^220^

KPT‐330 treatment had potent, dose‐dependent inhibitory effects on GIST cells. It effectively impeded cell proliferation by inducing G1‐phase arrest and triggering apoptosis. Furthermore, in three distinct GIST xenograft models, KPT‐330 successfully inhibited tumour growth and cell proliferation.^216^

In liposarcoma, KPT‐330 reduced tumour cell proliferation in vitro by initiating cell cycle arrest, promoting apoptosis and decreasing tumour size and volume in multiple xenograft models.^216^, ^220^, ^221^ Another study demonstrated that the antitumour impact of KPT‐330 treatment, when applied to three PDXs and two dedifferentiated liposarcoma tumour cell lines, was considerably greater than that of doxorubicin. This was evidenced by its superior tumour response and ability to induce apoptosis.^220^, ^222^ KPT‐330 treatment in conjunction with carfilzomib disrupted ribosome biogenesis networks or inhibited the pro‐survival factor prolin‐rich Akt substrate of 40 kD (PRAS40), thus decreasing tumour cell activity. Interestingly, the combined antitumour efficacy of KPT‐330 and carfilzomib appeared to be dose‐dependent. A low dose of carfilzomib combined with KPT‐330 has been shown to exhibit a superimposed effect, whereas a higher dose has demonstrated a synergistic effect.^220^, ^223^

In ASPS, although in vitro results suggested that two specific cell lines, ASPS‐KY and ASPS‐1, exhibited resistance to KPT‐330, compelling in vivo evidence demonstrated that KPT‐330 significantly hampered tumour growth and induced cell death in certain ASPS xenograft models.^216^ With respect to EWS, in vitro experiments demonstrated that KPT‐330 substantially inhibited most EWS cell lines. However, the A672 and CHP100 cell lines showed a lack of therapeutic sensitivity.^220^ Investigations further revealed that combining KPT‐330 with either carfilzomib or crizotinib (a c‐mesenchymal‐epithelial transition factor (c‐MET) inhibitor) markedly enhanced apoptosis in vitro, demonstrating superior efficacy, compared to monotherapy.^219^ The insulin‐like growth factor 1 (IGF1) receptor (IGF‐1R) has emerged as a promising therapeutic target for EWS. Interestingly, the knockdown of XPO1 expression resulted in increased expression of IGFBP3, an IGF1‐binding protein that regulates IGF signalling.^219^ Accordingly, the combination of KPT‐330 and linsitinib (an IGF‐1R inhibitor) effectively restrains tumour cell proliferation and has synergistic efficacy, compared to monotherapy.^219^

In osteosarcoma, KPT‐335 effectively inhibited cell proliferation, regardless of whether it was used independently or in combination with the proteasome inhibitor bortezomib (BORT). Additionally, KPT‐330 helps increase the sensitivity of tumour cells to radiation therapy, thereby augmenting the overall therapeutic impact of the treatment.^218^, ^220^, ^224^

### 6.10 Melanoma

Different SINEs, including KPT‐185, KPT‐276, KPT‐330 and KPT‐335, have been demonstrated to significantly inhibit the proliferation of melanoma cells and promote apoptosis in vitro.^225^, ^226^ Empirical evidence from in vivo studies has shown that treatment with KPT‐330 or KPT‐276 alone reduces tumour growth.^225^ Additionally, KPT‐330 can be utilised in combination with immunosuppressive agents, including programmed cell death protein 1 (PD‐1) inhibitors, programmed cell death protein 1 ligand 1 (PD‐L1) inhibitors and cytotoxic T‐lymphocyte‐associated protein 4 (CTLA4) inhibitors, for more effective melanoma treatment.^227^ However, the daily administration of KPT‐330 potentially leads to a diminished antitumour effect of combined treatment with KPT‐330 and PD‐L1 inhibitors.^228^ The strategic scheduling of treatment dosages may play a crucial role in enhancing melanoma immunotherapy efficacy. In addition, the concept of combination therapy holds promise for clinical treatment guidelines (Table 3).

**TABLE 3:** Summary of preclinical study of selective inhibitor of nuclear export (SINE) compounds efficacy in solid cancers in vitro and in vivo.

SINE	Cancer	Antitumour effect in vivo/in vitro	Effect of combination therapy in vivo/in vitro
KPT‐127	Pancreatic cancer	In vitro^272^	NR
	Prostate cancer	In vitro^309^	NR
KPT‐185	Lung cancer	In vitro^310^, ^311^	Cisplatin in vitro^190^; irinotecan in vitro^190^
	Triple‐negative breast cancer	In vitro^95^, ^312^	NR
	Gastric cancer	In vitro^178^, ^313^	NR
	Pancreatic cancer	Both^272^, ^314^	NR
	Ovarian cancer	In vitro^315^	NR
	Kidney cancer	In vitro^316^	NR
	Prostate cancer	In vitro^177^	NR
	Melanoma	In vitro^317^	NR
KPT‐205	Pancreatic cancer	In vitro^272^	NR
	Prostate cancer	In vitro^309^	NR
KPT‐225	Prostate cancer	In vitro^309^	NR
KPT‐227	Pancreatic cancer	In vitro^272^	NR
KPT‐251	Glioblastoma	In vitro^182^	NR
	Triple‐negative breast cancer	In vitro^95^	NR
	Pancreatic cancer	Both^314^	NR
	Kidney cancer	Both^316^	NR
	Prostate cancer	Both^177^, ^309^	Docetaxel (DTX) in vitro and vivo^318^
	Diffuse malignant peritoneal mesothelioma	Both^319^	Survivin inhibitor in vitro^319^
	Melanoma	Both^317^	PLX‐4032, a BRAF inhibitor, in vitro
KPT‐276	Glioblastoma	Both^182^	NR
	Triple‐negative breast cancer	In vitro^95^	NR
	Non‐small cell lung cancer	In vivo^311^	NR
	Gastric cancer	In vivo^313^	NR
	Diffuse malignant peritoneal mesothelioma	Both^319^	Survivin inhibitor in vitro^319^
	Melanoma	Both^317^	PLX‐4720, a BRAF inhibitor in vitro and vivo^317^
KPT‐301	Prostate cancer	In vitro^177^	NR
KPT‐330	Glioblastoma	Both^182^	Bcl‐xL/Bcl‐2 inhibitor in vitro and vivo^320^; Irradiation in vitro and in vivo^186^; olaparib, a poly(ADP‐ribose) polymerase (PARP) inhibitor in vitro and vivo^321^
	Lung cancer	Both	Enzalutamide in vivo^322^; cisplatin in vitro and vivo^190^, ^322^; irinotecan in vitro and vivo^190^; KRAS G12C inhibitor in vitro and vivo^191^; PARP1 inhibition in vitro and vivo^189^; Bcl‐xL inhibitor in vitro and vivo^184^
	Breast cancer	Both^95^, ^312^	Tamoxifen in vitro and vivo^134^; olaparib, a PARP inhibitor in vitro and vivo^199^; LOM612, a small molecule activator of FOXO nuclear‐cytoplasmic shuttling in vitro and in vivo^323^; a PI3K/mTOR inhibitor in vitro and in vivo^197^; tucidinostat in vitro and vivo^201^; Y219, a novel proteasome inhibitor in vitro and vivo^324^
	Gastric cancer	Both^178^, ^313^	Nab‐paclitaxel in vitro and vivo^178^
	Pancreatic cancer	Both	Gemcitabine in vitro and vivo^146^; gemcitabine plus nab‐paclitaxel in vitro and vivo^147^, ^233^
	CRC	Both	Bortezomib (BORT) in vitro and vivo^204^; irradiation in vitro and in vivo^207^; ATR inhibitors in vitro^205^
	Cervical cancer	In vitro	A Bcl‐xL inhibitor in vitro^184^
	Ovarian cancer	Both^315^	Decitabine in vitro^325^; an inhibitor of MDM2‐p53 interaction in vitro^209^; cisplatin in vitro and vivo^315^, ^326^; olaparib in vitro and vivo^327^
	Prostate cancer	Both^309^	Abiraterone in vitro^214^; enzalutamide in vitro and vivo^214^, ^322^; DTX in vitro and vivo^318^; cisplatin in vitro and vivo^322^
	Sarcoma	Both	BORT in vitro and vivo^328^
	Gastrointestinal stromal tumours	Both^216^	Imatinib in vitro^216^
	Liposarcoma	Both^221^	Carfilzomib in vitro^329^
	Dedifferentiated liposarcoma	Both^216^	NR
	Sarcoma of soft part of alveolar	Both^216^	NR
	Ewing sarcoma	Both	Crizotinib, a c‐MET inhibitor, in vitro^219^; linsitinib in vitro and vivo^219^
	Osteosarcoma	In vitro	Irradiation in vitro^330^
	Leiomyosarcoma	Both^216^	NR
	Uterine leiomyosarcoma	Both	Doxorubicin/gemcitabine in vitro and vivo^331^
	Rhabdomyosarcoma	In vitro^216^	NR
	Undifferentiated sarcomas	Both^216^	Palbociclib in vitro and in vivo^332^
	Diffuse malignant peritoneal mesothelioma	Both^319^	Survivin inhibitor in vitro^319^
	Melanoma	Both^317^	PLX‐4720, a BRAF inhibitor in vivo^317^; anti‐CTLA4/anti‐PD‐1/anti‐PD‐L1 antibodies in vivo^333^
KPT‐335	Esophageal cancer	Both^334^	NR
	Neuroblastoma	Both^264^	NR
KPT‐8602	Gastric cancer	In vitro^178^	NR
	Hepatocellular carcinoma	Both	Sorafenib in vivo^335^
	CRC	Both	ATR inhibitors in vitro and vivo^205^
	Prostate cancer	Both	PARP inhibitors in vitro^336^; abiraterone in vivo^214^; enzalutamide in vitro^214^

Abbreviations: FOXO, forkhead box protein O; NR, not reported.

## 7 CLINICAL TRIALS OF XPO1 INHIBITORS

Given the noteworthy therapeutic effectiveness of SINEs in preclinical trials for solid tumours, a growing number of comprehensive clinical studies have investigated the impacts of selinexor, either as a standalone treatment or in combination with other agents. Table 4 provides an overview of completed studies that have contributed to our understanding of the efficacy of selinexor in treating solid tumours. To date, most clinical trials of the application of selinexor in solid tumours are still in the recruitment phase. This suggests that the promising potential of KPT‐330 for treating solid tumours warrants further exploration to establish a safe, effective and rational treatment plan. To date, KPT‐330 has achieved incremental success in clinical trials across several types of solid tumours, underscoring its potential role in advancing cancer therapy.

**TABLE 4:** Summary of clinical trial results on selinexor's efficacy in solid cancers.

NCT	Trial	Completed or not	Population and tumour type	Selinexor and other treatment	Outcomes	Haematopoietic‐related adverse events
NCT01896505	Phase Ib trial^337^	Completed	54 patients with advanced refractory bone or soft tissue sarcoma	30 mg/m^2^, 50 mg/m^2^ or flat dose of 60 mg	33% (17/52) had SD for ≥4 months	Grade 3 or 4 toxicities were thrombocytopenia (7.7%), anaemia (9.7%), lymphopenia (9.7%) and leukopenia (9.7%)
NCT02215161	Phase II trial^338^	No	14 patients in abiraterone‐ and/or enzalutamide‐refractory metastatic castration‐resistant prostate cancer	65 mg/m^2^ BIW and subsequently 60 mg/m^2^ BIW	14.2% (2/14) had PR and 28.6% (4/14) had SD	Grade 3 or 4 toxicities were anaemia (21%), neutrophil count decreased (7%), hypophosphatemia (7%)
NCT02419495	Phase Ib trial^339^	No	13 patients with advanced solid tumours	Selinexor 60 mg BIW or 60–80 mg QW + carboplatin with paclitaxel	7.7% (1/13) had PR and 38.5% (5/13) had SD	Grade 3 or 4 toxicities were neutropenia (85%), leukopenia (85%), thrombocytopenia (85%), anaemia (69%)
NCT03042819	Phase Ib trial^340^	Completed	25 patients with advanced soft tissue sarcomas	Selinexor 60 mg OR 80 mg QW; doxorubicin 75 mg/m^2^ Q3W	20.8% (5/24) had PR, and 62.5% (15/24) had SD	Grade 3 or 4 toxicities were neutropenia (56%), febrile neutropenia (28%) and anaemia (24%)
NCT02178436	Phase Ib trial^341^	Completed	9 patients with pancreatic ductal adenocarcinoma	Selinexor QW; GEM 1000 mg/m^2^ QW and nab‐paclitaxel 125 mg/m^2^ QW	40% (2/5) had PR and 40% (2/5) had SD	Grade 3 or 4 toxicities were anaemia, thrombocytopenia, leukopenia and lymphopenia
NCT01607905	Phase I trial^342^	Completed	189 patients with advanced solid tumours	Selinexor dose escalation: 3–85 mg/m^2^	0.7% (1/152) had CR, 3.9% (6/152) had PR and 17.8% (27/152) had SD for ≥4 months	Grade 3 or 4 toxicities were thrombocytopenia (16%) and hyponatremia (13%)
NCT02269293	Phase I trial^343^	Completed	23 patients with advanced ovarian or endometrial cancers (ECs)	1 of 4 alternating regimens: 1. Selinexor 30 mg/m^2^ BIW, carboplatin AUC 5 IV, paclitaxel 175 mg/m^2^ IV Q3W; 2. Selinexor 30 mg/m^2^ BIW, carboplatin AUC 5 IV, paclitaxel 80 mg/m^2^ IV QW; 3. Selinexor 60 mg/m^2^ QW, carboplatin AUC 5 IV, paclitaxel 80 mg/m2 IV QW; 4. Selinexor 60 mg/m^2^ QW, carboplatin AUC 5 IV, paclitaxel 175 mg/m^2^ IV Q3W	4.3% (1/23) had CR, 52.2% (12/23) had PR and 13% had SD	Grade 3 or 4 toxicities were leukopenia (70%), neutropenia (70%), lymphopenia (61%) and anaemia (57%)
NCT02419495	Phase Ib trial^344^	No	14 patients with advanced or metastatic solid tumours	Selinexor 60 mg BIW or 60–80 mg QW + topotecan 0.5 to 1.5 mg/m^2^ daily for 5 days	46.2% (6/13) had SD	Grade 3 or 4 were anaemia (21 %), neutropenia (21 %), thrombocytopenia (21 %) and leukopenia (14 %)
NCT02025985	Phase II trial^345^	Completed	114 patients with recurrent gynaecological malignancies	35 or 50 mg/m^2^ BIW or 50 mg/m^2^ QW	11.2% (11/98) had PR and 23.5% (23/98) had SD	Grade 3 or 4 were thrombocytopenia (17%) and anaemia (10%)
NCT02419495	Phase Ib trial^346^	No	35 patients with metastatic solid tumours	Selinexor 60 or 80 mg BIW + paclitaxel 80 mg QW	12.9% (4/31) had PR and 32.3% (10/31) had SD	Grade 3 or 4 were neutropenia (46%) and anaemia (31%)
NCT02606461	Phase II‐III trial^347^	Completed	285 patients with dedifferentiated liposarcoma	Selinexor 60 mg BIW or placebo BIW	2.66% (5/188) had PR and 59.0% (111/188) had SD	Grade 3 or 4 were anaemia (18.7%), thrombocytopenia (10.2%) and neutropenia (9.1%)
NCT03555422	Phase III trial^348^	No	263 patients with advanced or recurrent EC	Selinexor 80 mg QW or placebo QW	NR	Grade 3 or 4 were neutropenia (8.8%), thrombocytopenia (7.0%), anaemia (2.3%) and leukopenia (1.2%)
NCT02419495	Phase Ib trial^349^	No	31 patients with advanced solid tumours and triple‐negative breast cancer	Two stages: 1. Selinexor 60 mg BIW, eribulin 1 mg/m^2^ QW; 2. Selinexor 80 mg QW, eribulin 1 mg/m^2^ QW;	9.7% (3/31) had PR and 58.1% (18/31) had SD	Grade 3 or 4 were neutropenia (29%), leukopenia (26%) and anaemia (16%)
NCT02384850	Phase I trial^350^	No	10 patients with metastatic CRC	Selinexor 20 or 40 mg + 5‐fluorouracil, leucovorin and oxaliplatin (mFOLFOX6)	NR	No obvious grade 3 or 4 were observed
NCT02137356	Phase I trial^351^	No	11 patients with locally advanced rectal cancer	Selinexor 20 mg/m^2^ BIW + chemoradiation, or 35 mg/m^2^ BIW+ chemoradiation, or 35 mg/m^2^ BIW + 2 weeks after chemoradiation	NR	No obvious grade 3 or 4 were observed
NCT02402764	Phase II trial^352^	Completed	10 patients with metastatic triple‐negative breast cancer	60 mg BIW	30% (3/10) had SD	Grade 3 or 4 was platelet count decreased (10%)
NCT01986348	Phase II trial^353^	No	76 patients with recurrent glioblastoma	Arm B: 50 mg/m^2^ BIW	Arm B: 8.3% (2/24) had PR, 25% (6/24) had SD	Grade 3 or 4 were thrombocytopenia (4.4%), leukopenia (5.9%), neutropenia (11.8%) and lymphopenia (5.9%)
				Arm C: 60 mg flat dose BIW	Arm C: 7.7% ( 1/13) had PR, 30.8% (4/13) had SD	
				Arm D: 80 mg flat dose QW	Arm D: 3.3% (1/30) had CR, 6.7% (2/30) had PR, 23.3% (7/30) had SD	
**NR**	Phase I trial^354^		22 patients with advanced CRC	4 schedules	WT: 12.5% (1/8) had disease control rate (DCR) of >3 months	NR
				1: 40 mg/m^2^ BIW until 28 days	RAS/AKT‐mut: 40% (4/10) had DCR of >3 months	
				2: 50 mg/m^2^ QW continuous until 28 days		
				3: 40 mg/m^2^ BIW for 2 weeks, until 21 days		
				4: 20 mg/m^2^ TIW until 28 days		
NCT02120222	Phase I trial^355^	Completed	7 patients with unresectable melanoma	50 mg/m^2^ BIW	14.3% (1/7) had a minor response, 57.2% (4/7) experienced SD	Grade 3 or 4 were neutropenia (14.3%), thrombocytopenia (42.9%), anaemia (14.3%)
NCT04256707	Phase I/II trial^356^	No	29 patients with CRC	Selinexor 80 mg QW and pembrolizumab 200 mg IV every 3 weeks	38.9% (7/18) had SD	NR
NCT02078349	Phase I trial	Completed	120 patients with advanced or metastatic solid tumour malignancies	3 schedules	NR	NR
				1:50 mg/m^2^ QW continuous until 28 days		
				2:40 mg/m^2^ BIW continuous until 21 days		
				3:20 mg/m^2^ TIW continuous until 28 days		
NCT02250885	Phase II trial	Completed	14 patients with poorly differentiated lung and gastrointestinal and pancreatic neuroendocrine tumours	50 mg/m^2^ BIW on weeks 1, 2 and 3 of each 4‐week cycle	NR	NR
NCT03095612	Phase I/II trial	Completed	40 patients with NSCLC	Selinexor QW or BIW and DTX Q3W (75 mg/m^2^ IV)	NR	NR

Abbreviations: BIW, twice a week; CR, completed response; NR, not reported; NSCLC, non‐small cell lung cancer; PR, partial response; Q3W, thrice a week; QW, once a week; SD, stable disease; TIW, thrice a week.

*Source*: All data are from the US National Institutes of Health (NIH) clinical trial database (clinicaltrials.gov).

### 7.1 Glioblastoma

In a phase II clinical study (NCT01986348) of the use of oral selinexor for the treatment of glioblastoma, 76 patients were enrolled. Although varying degrees of treatment‐related adverse events were reported with the use of oral selinexor, these adverse effects were mitigated by adjusting the drug dosage.^229^ For the nonsurgical treatment cohort (*n* = 68), 28% (19/68) of the tumours exhibited a significant reduction in tumour size. Additionally, this group also reported an objective response rate (ORR) of 10% (3/30), and the highest 6‐month PFS rate of 17.7% was noted in the oral 80 mg/once weekly treatment subgroup. This subgroup was unique because it achieved a complete response (3.3%, 1/30) and two instances of PR (6.7%, 2/30). In contrast, in the surgical treatment cohort (*n* = 8), immunohistochemistry indicated that selinexor treatment effectively reduced cell proliferation (Ki67: pretreatment vs. posttreatment 29% ± 3% vs. 13% ± 0.8%, *p* = .012) and promoted apoptosis (cleaved caspase‐3: pretreatment 2% ± 0.7% vs. 28% ± 3.0%, *p* = .003). Remarkably, one patient continued oral selinexor treatment for up to 42 months. The promising efficacy and safety outcomes observed in this trial suggest that further exploration of the potential of selinexor for treating glioblastoma is warranted.

### 7.2 Breast cancer

In a phase II clinical study (NCT02402764) of selinexor administration for the treatment of metastatic TNBC (*n* = 10), patients were orally administered 60 mg of selinexor twice a week, during which the drug was well tolerated.^230^ However, the combined efficacy—considering completed response (CR), PR and stable disease (SD)—was rather limited at only 30% (three out of 10 patients). In addition, both in vivo and in vitro studies have shown that combined treatment has a more substantial therapeutic effect on tumours than monotherapy.^195^, ^196^, ^197^, ^198^ Despite these findings, the study was unfortunately terminated due to interim results failing to meet expectations. Nevertheless, the results underscore the need for additional clinical trials involving selinexor in combination with other drugs for breast cancer treatment. In another study (NCT02419495), selinexor in combination with paclitaxel was administered to two breast cancer patients. The results showed SD in 50% of the patients (one out of 2 patients) and PD in the other 50% (one out of 2 patients).^231^

### 7.3 Pancreatic cancer

According to a study regarding the use of selinexor for the treatment of advanced solid tumours in Asia, patients with solid malignancies with PI3K/AKT/rat sarcoma (RAS) pathway mutations had a median PFS of 40 days, whereas the PFS was 32 days for the other six pancreatic cancer patients.^232^ Furthermore, the study succeeded in establishing the optimal dose of selinexor for the forthcoming phase 2 study, setting the stage for subsequent efficacy validation. To further evaluate the substantial clinical efficacy of selinexor in combination with gemcitabine and nab‐paclitaxel, a total of nine patients were enrolled in this study (NCT02178436).^233^ Over the course of the research period, 22.2% (2/9) of the patients treated with this combination achieved a PR, while another 22.2% (2/9) achieved SD. Remarkably, one patient even achieved a 16‐month PFS following treatment, culminating in an OS time of 22 months. These findings underscore the promising potential of selinexor for the treatment of pancreatic cancer.

### 7.4 CRC

In the first clinical study (NCT01607905) regarding the use of selinexor for the treatment of advanced solid tumours (*n* = 189), histochemical analyses performed approximately 4 weeks before and after selinexor treatment revealed that selinexor reduced the number of tumour cells by promoting apoptosis and reducing tumour cell proliferation.^10^ Furthermore, one patient showed PD in imaging studies. In a subset of the study, biopsies were taken from 43 patients with CRC, revealing that 29 patients had *KRAS* mutations. Among the patients with *KRAS* mutations, 44% exhibited SD, whereas only 12% of the patients in the KRAS WT group exhibited SD. A subsequent phase I trial examining the impact of selinexor on patients with advanced CRC harbouring either WT or RAS/AKT pathway mutations aimed at evaluating treatment efficacy.^234^ Interestingly, PFS was longer in patients with RAS/AKT pathway mutations than in those with WT RAS/AKT pathway expression (78 days vs. 50 days). Moreover, the disease control rate (DCR) was greater in patients with RAS/AKT mutations (four out of 10 or 40%) than in WT patients (one out of eight or 12.5%). Finally, in a recent study assessing selinexor tolerance in Asian patients with advanced solid tumours (*n* = 14), the median PFS was reported to be 42 days.^232^

To enhance our understanding of the safety and tolerability of selinexor in combination with calcium 5‐fluorouracil, folinic acid and oxaliplatin (mFOLFOX6) for treating metastatic CRC, a phase I clinical trial (NCT02384850) was conducted with 10 patients.^235^ Regrettably, the trial encountered a setback when dose‐limiting toxicity necessitated the withdrawal of four patients. Even after the selinexor dose was reduced, another four patients independently chose to withdraw from the study following the first treatment cycle. Moreover, the treatment's effectiveness was insufficient to achieve the anticipated clinical outcomes. For future investigations, adjusting the drug combination and treatment dosage regimen may be beneficial. Previous animal research has indicated that treatment with selinexor augments sensitivity to radiotherapy in mouse xenograft models.^206^, ^207^ A new phase I clinical trial (NCT02137356) enrolled 11 patients with rectal cancer to study the efficacy and safety of different doses of selinexor (20, 35, 50 mg/m^2^) in combination with neoadjuvant radiotherapy.^236^ However, two patients from the highest dose group opted out of the trial due to poor tolerance of subsequent treatments. Of the nine remaining patients, two exhibited a PR, achieving a near complete or complete pathologic response. For the optimal delineation of an appropriate selinexor dose in conjunction with neoadjuvant radiotherapy, a phase II clinical trial is advised. Furthermore, a phase I open‐label study (NCT04256707) investigated the efficacy of selinexor combined with pembrolizumab for patients (*n *= 29) resistant to chemotherapy in CRC.^237^ Of the 18 evaluated patients, 38.9% (7/18) had SD, and 83.3% (6/7) of those with SD had *RAS* mutations. This study underscores the effectiveness of selinexor in combination with pembrolizumab in combating chemotherapy‐resistant CRC. Given the satisfactory safety profile of this combination, further exploration of CRC treatment modalities could be conducted, particularly focused on *RAS*‐mutant CRC.

### 7.5 Prostate cancer

In an initial clinical investigation (NCT01607905) regarding the use of selinexor for the treatment of advanced solid tumours (*n* = 189), histochemical analysis was performed approximately 4 weeks prior to and after selinexor treatment. Treatment with selinexor noticeably reduced the tumour cell count by inducing apoptosis and curtailing tumour cell proliferation.^10^ Concurrently, PD was observed by imaging in one patient with prostate cancer. Regrettably, among patients who withdrew from the study, two had prostate cancer and had SD for 325 and 72 days before any signs of disease progression were identified. In a separate clinical investigation (NCT02215161) involving metastatic debulking‐resistant prostate cancer patients (*n* = 14), the efficacy of selinexor in combination with enzalutamide and abiraterone was examined.^238^ Due to poor drug tolerance, 21% (3/14) of the patients had to prematurely discontinue their participation in the study. Furthermore, severe adverse effects were reported by 36% (5/14) of the patients, leading to early termination of the study. Of the eight patients who could be evaluated, 50% had SD, 25% had PR and some had a decreasing trend in prostate‐specific antigen (PSA) levels after selinexor treatment. These findings underscore the necessity of continued investigation into the clinical implications of selinexor for the treatment of prostate cancer.

### 7.6 Gynaecologic oncology

In an initial clinical study (NCT01607905), the efficacy of selinexor on solid tumours was evaluated; one patient with ovarian cancer (1/8) and one patient with cervical cancer (1/7) achieved a PR as demonstrated by posttreatment imaging.^10^ In a multicentre phase I study, the safety, tolerability and efficacy of selinexor were evaluated in patients with advanced platinum‐resistant ovarian cancer.^111^ The findings showed that selinexor therapy hindered tumour growth in 60% (3/5) of the evaluated patients. In five evaluable patients, selinexor treatment inhibited tumour growth in 60% (3/5) of patients, with one showing a PR and the other two demonstrating SD and moderate tolerability of the drug. In a subsequent phase II study (NCT02025985), the efficacy and safety of selinexor were examined for recurrent gynaecologic tumours. Phase I included 25 ovarian cancer patients, 23 endometrial cancer (EC) patients and 25 cervical cancer patients, while phase II included 41 ovarian cancer patients.^239^ Following phase I treatment, the DCR was 28% for ovarian cancer, 24% for cervical cancer and 35% for EC. After phase II treatment, the DCR was 33% for patients with ovarian cancer, with a selinexor treatment dose of 35 mg/m^2^ administered twice weekly and 50 mg/m^2^ once weekly. Additionally, eight patients achieved a PR throughout the treatment period. In summary, this research demonstrated that selinexor, when administered orally, has a modest degree of efficacy in the management of ovarian and ECs.

To enhance our understanding of the effectiveness and safety of selinexor combined with chemotherapy for treating gynaecological tumours, a study (NCT02419495) included 14 patients suffering from various gynaecological malignancies.^240^ The group comprised patients with various gynaecological tumours, including ovarian cancer (*n* = 5), EC (*n* = 2) and single instances of both fallopian tube and vaginal cancer. Following treatment with a 60 mg dose of selinexor coupled with topotecan, a promising 46% of patients (6/13) exhibited SD and demonstrated moderate tolerance to the medication. These findings suggest the potential of this combined therapeutic approach for gynaecological cancer treatment. Furthermore, an additional phase I clinical study (NCT02269293) involving five patients with ovarian cancer and 18 patients with EC evaluated the safety and effectiveness of selinexor in combination with paclitaxel and carboplatin.^241^ Although the sample size for this study was somewhat limited, the results were encouraging, with a notable treatment effect observed in 56.5% (13/23) of the patients. These clinical studies underscore the potential therapeutic effectiveness of selinexor combined with chemotherapy for treating gynaecological cancers. In a randomised, prospective, multicentre, double‐blind, placebo‐controlled, phase III study (NCT03555422), 263 patients with advanced or recurrent EC were randomly assigned to receive selinexor or placebo after combination chemotherapy.^242^ Notably, patients with *p53* WT EC had a median PFS of 13.7 months when treated with selinexor and 3.7 months when treated with placebo. These findings suggest that selinexor is important for maintenance therapy in *p53* WT ECs, but this requires further investigation.

In a single‐centre, multiarm phase Ib clinical trial (NCT02419495), 28 patients with refractory or platinum‐resistant ovarian cancer were enrolled, along with one individual with cervical cancer.^231^ Almost all participants experienced a treatment‐emergent adverse event (TEAE). However, these findings were all within an assessable range. The most common TEAEs of grade ≥ 3 included neutropenia (46%), anaemia (31%) and nausea (21%). Among the 24 patients with ovarian cancer that were evaluated, a response rate of 17% (4/24), a clinical benefit rate of 58% (14/24) and a median PFS of 6.8 months were observed. This evidence of the modest clinical efficacy and manageable toxicity of these agents suggests that further exploration of combination therapy for malignant gynaecologic tumours could be beneficial.

### 7.7 Sarcoma

In a phase Ib clinical trial (NCT01896505) designed to assess treatment efficacy and safety, 54 patients with advanced, refractory bone/soft tissue sarcoma were treated with various doses of selinexor (30, 50, or 60 mg/m^2^).^243^ Despite the absence of CR or PR among the 52 evaluable patients, SD was observed for 4 months or longer in 33% (17/52) of patients. This included seven patients with dedifferentiated liposarcoma (out of 15), 40% (6/15) of whom exhibited significant antitumour effects. In a subsequent phase II‐III randomised, multicentre, double‐blind, placebo‐controlled study (NCT02606461), 285 patients with advanced refractory dedifferentiated liposarcoma were enrolled (188 on selinexor, 97 on placebo).^244^ PFS increased by 30% after 12 or more weeks of selinexor treatment, compared to that in the placebo group, with an ORR of 2.7% (5/188). However, after a median follow‐up of 14.6 months, OS did not significantly differ between the selinexor group and the placebo group. These findings provide innovative therapeutic insights for the treatment of dedifferentiated liposarcoma.

In a phase Ib trial (NCT03042819), selinexor was tested in combination with adriamycin for treating patients with advanced soft tissue sarcoma.^245^ A total of 25 patients were included in the trial. Eighty percent of these patients experienced grade 3 or higher treatment‐related adverse events (AEs). The most prevalent haematologic adverse events included anaemia, leukopenia, neutropenia and granulocytopenia, each affecting 92% of the participants. Despite these adverse effects, 21% of patients achieved a PR, while 63% achieved SD, leading to an overall DCR of 84%. This finding suggests the potential applicability of combining selinexor with adriamycin for soft tissue sarcoma treatment. Furthermore, the effects of the administration of 80 mg of selinexor in combination with adriamycin were investigated. This study also revealed possibilities for incorporating other drugs to mitigate the associated toxic side effects.

### 7.8 Melanoma

In an initial clinical trial (NCT01607905) investigating the use of selinexor for solid tumours (*n* = 189), CR was observed in one melanoma patient, while another demonstrated PR, as revealed by imaging.^10^ A separate study (NCT02120222) focused on unresectable melanoma involving seven patients demonstrated that 57.1% (4/7) of patients achieved SD and 14.3% (1/7) experienced a minor response. This led to a DCR of 71.4% (5/7).^246^ Due to the favourable tolerability of selinexor among melanoma patients, another trial was performed. The results from this subsequent melanoma study are anticipated (NCT02120222).

## 8 SAFETY AND ADVERSE EFFECTS IN PATIENTS WITH SOLID CANCERS

Given the remarkable therapeutic effect of selinexor on haematologic malignancies, the question remains whether selinexor has toxic effects on haematopoietic stem cells in solid tumours. In a phase I clinical trial (NCT01607905) of selinexor for advanced solid tumours, the most common haemosystem‐related AEs were thrombocytopenia (16%), anaemia (9%) and neutropenia (8%).^10^ Most AEs, including fatigue, nausea, anorexia, vomiting and hyponatremia, were manageable with the indicated agents. Notably, the occurrence of hyponatremia includes multiple factors (such as comorbidities and medications) and is often associated with selinexor‐related gastrointestinal side effects (such as nausea and vomiting).^247^ Other studies have shown that the unique mechanism of selinexor and its effective penetration into the central nervous system may lead to gastrointestinal AEs.^182^, ^248^ Recently, eltanexor has been tested in clinical trials for the treatment of haematologic malignancies.^249^ Compared to selinexor, eltanexor reduced the incidence and severity of hyponatremia and decreased appetite, fatigue and nausea in MM patients.^250^ Subsequent studies have shown that haematological AEs can be controlled by reducing selinexor doses or adding the indicated agents.^229^, ^231^ For example, inhibition of XPO1 expression leads to thrombocytopenia by inhibiting thrombopoietin (TPO) signalling and blocking the differentiation of stem cells into megakaryocytes.^251^ TPO agonists reverse selinexor‐induced thrombocytopenia. In a phase 1 clinical trial regarding the use of selinexor combined with neoadjuvant chemoradiation for the treatment of patients with locally advanced rectal cancer, thrombocytopenia resolved within days of pausing treatment.^236^

## 9 RESPONSE BIOMARKERS

**FIGURE 5:**
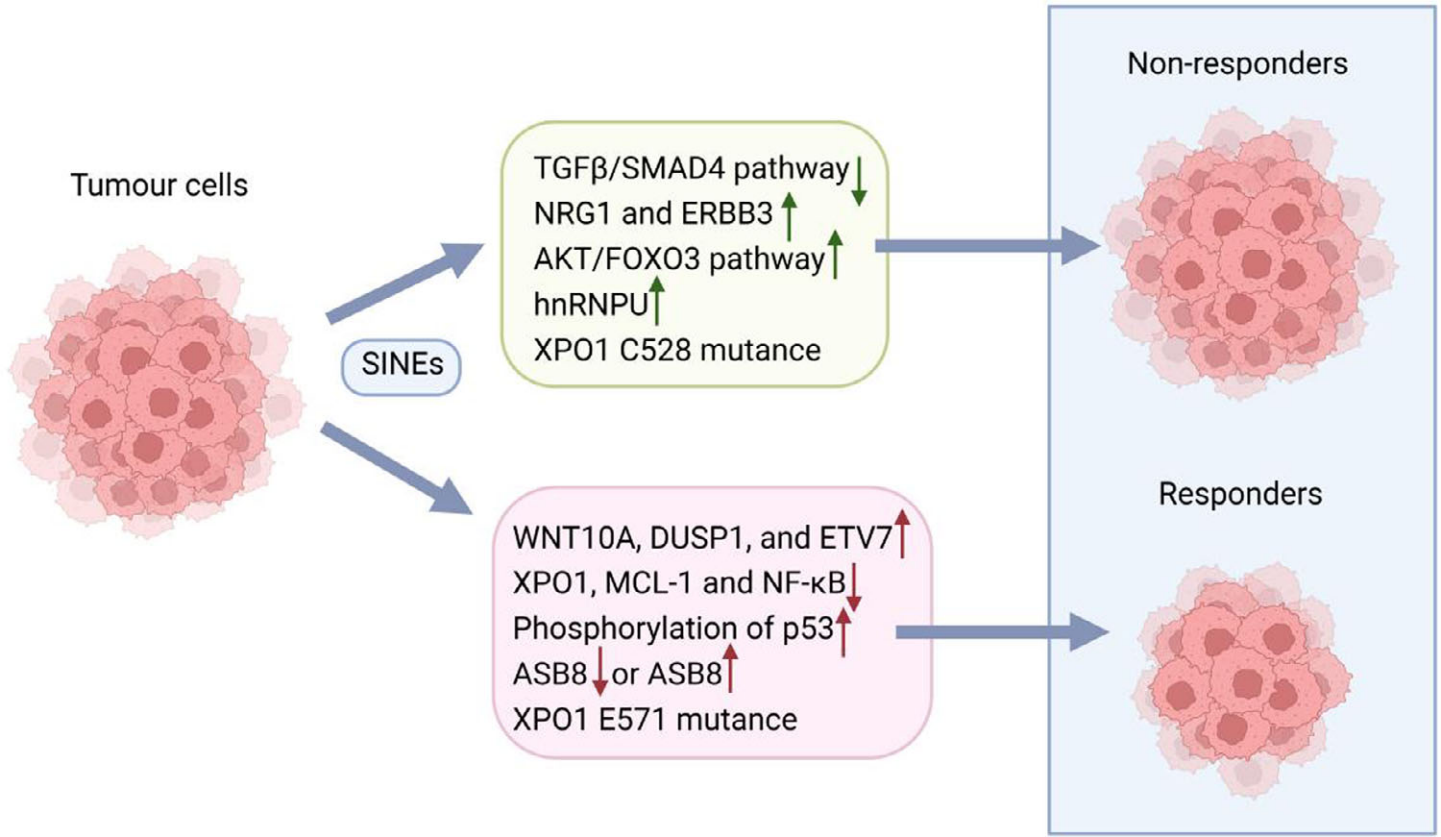
Schematic diagram of response biomarkers of selective inhibitor of nuclear exports (SINEs) in tumour therapy.

Although XPO1 inhibitors have demonstrated potent antitumour effects, not all patients benefit from XPO1 inhibitors. *XPO1* mutations, especially the *XPO1* E571 mutation, occur in a variety of cancers.^36^, ^252^ The *XPO1* E571 mutation promotes the development of B‐cell malignancies and increases therapeutic sensitivity to selinexor.^33^ Similarly, the *XPO1* E571 mutation was detected in plasma cell‐free DNA from Hodgkin's lymphoma patients and was associated with tumour regression or progression. Upregulation of neuregulin 1 (NRG1) and ERBB3 expression inhibits the antitumour effect of SINEs.^211^ In MM, downregulation of the activity of the transforming growth factor‐β (TGFβ)/drosophila mothers against decapentaplegic protein 4 (SMAD4) pathway was associated with resistance to selinexor.^253^ Inhibition of the RNA‐binding protein (hnRNPU) increased the sensitivity of selinexor treatment.^254^ Interestingly, both ankyrin repeat and the suppressor of cytokine signalling (SOCS) box containing 8 overexpression and knockout sensitise cells to selinexor.^253^ A study found that selinexor upregulates the expression of P2RY2 (a purinergic receptor), which leads to the activation of the AKT pathway.^255^ Activation of the AKT pathway, which promotes FOXO3a nuclear export, limits the efficacy of selinexor, and the combination of AKT inhibitors and selinexor increases the efficacy.^256^ A novel three‐gene expression signature (*WNT10A*, *DUSP1* and *ETV7*) predicts selinexor response in MM patients. High expression of these three genes was associated with prolonged PFS in MM patients treated with selinexor, suggesting that the expression signatures of these three genes are potential biomarkers for predicting treatment outcomes.^257^ A recent study showed that XPO1, nuclear factor kappa B (NF‐κB) (p65), myeloid cell leukemia‐1 (MCL‐1) and p53 protein levels in haematological tumours may be used as biomarkers of the response to XPO1 inhibitor therapy.^258^ However, due to the small sample size, verification with a larger sample size is needed. Furthermore, p53 phosphorylation was found to be a key regulator of selinexor treatment efficacy.^256^ Notably, at present, there are few therapeutic options for solid tumours treated with SINEs. The therapeutic predictors for solid tumours treated with SINEs need to be verified and expanded upon by further research (Figure 5).

## 10 CONCLUSION

XPO1, a critical component of nuclear‐cytoplasmic transport, plays a pivotal role in tumour development and therapeutic resistance. The targeted disruption of the nuclear‐cytoplasmic transport functionality of XPO1 has led to promising outcomes in cancer therapy.

In clinical trials for solid tumours, nuclear export‐selective inhibitors have shown notable antitumour activity via multiple pathways. The potential of these agents coupled with targeted drugs, chemotherapeutic agents and cycle inhibitors enhances their effectiveness. Moreover, combination treatment for solid tumours could reverse drug resistance to a certain degree. Furthermore, the FDA approved selinexor, an XPO1 inhibitor, for MM and refractory diffuse large B‐cell lymphoma. Despite potential concerns surrounding the safety and feasibility of XPO1 inhibitors in solid tumours, preclinical studies have revealed remarkable antitumour activity. The combination of these inhibitors with various existing drugs has yielded encouraging results, indicating the need for a more thorough investigation into the use of XPO1 inhibitors for solid tumour treatment. Notably, nuclear export‐selective inhibitors bind covalently, causing XPO1 inhibition. However, Cys528 mutations have never been detected in humans treated with XPO1 inhibitors. Gene editing technology has revealed that the Cys528 mutation can inactivate these inhibitors.^259^, ^260^ This finding suggests the continuous need for the optimisation of nuclear export‐selective inhibitors.

In conclusion, XPO1 targeting holds considerable promise for solid tumour treatment. Nuclear export‐selective inhibitors such as selinexor have shown significant efficacy in treating solid tumours and reducing drug resistance, suggesting new therapeutic directions for future treatments. To optimise the effectiveness of these agents in managing solid tumours, nuclear export‐selective inhibitors should be further refined through extensive clinical trials.

## AUTHOR CONTRIBUTIONS

Chuanxi Lai drafted the manuscript. Lingna Xu and Shengdai discussed and revised the manuscript. All the authors read and approved the final manuscript.

## CONFLICT OF INTEREST STATEMENT

The authors declare no conflicts of interest.

## Data Availability

No data were generated for the research described in the article.
